# Evaluation of the Effects of Erythritol on Gene Expression in *Brucella abortus*

**DOI:** 10.1371/journal.pone.0050876

**Published:** 2012-12-14

**Authors:** María Cruz Rodríguez, Cristina Viadas, Asunción Seoane, Félix Javier Sangari, Ignacio López-Goñi, Juan María García-Lobo

**Affiliations:** 1 Departamento de Biología Molecular, Instituto de Biomedicina y Biotecnología de Cantabria (IBBTEC), Universidad de Cantabria-CSIC-SODERCAN, Santander, Cantabria, Spain; 2 Departamento de Microbiología y Parasitología, Universidad de Navarra, Pamplona, Navarra, Spain; East Carolina University School of Medicine, United States of America

## Abstract

Bacteria of the genus *Brucella* have the unusual capability to catabolize erythritol and this property has been associated with their virulence mainly because of the presence of erythritol in bovine foetal tissues and because the attenuated S19 vaccine strain is the only *Brucella* strain unable to oxydize erythritol. In this work we have analyzed the transcriptional changes produced in *Brucella* by erythritol by means of two high throughput approaches: RNA hybridization against a microarray containing most of *Brucella* ORF's constructed from the *Brucella* ORFeome and next generation sequencing of *Brucella* mRNA in an Illumina GAIIx platform. The results obtained showed the overexpression of a group of genes, many of them in a single cluster around the *ery* operon, able to co-ordinately mediate the transport and degradation of erythritol into three carbon atoms intermediates that will be then converted into fructose-6P (F6P) by gluconeogenesis. Other induced genes participating in the nonoxidative branch of the pentose phosphate shunt and the TCA may collaborate with the *ery* genes to conform an efficient degradation of sugars by this route. On the other hand, several routes of amino acid and nucleotide biosynthesis are up-regulated whilst amino acid transport and catabolism genes are down-regulated. These results corroborate previous descriptions indicating that in the presence of erythritol, this sugar was used preferentially over other compounds and provides a neat explanation of the the reported stimulation of growth induced by erythritol.

## Introduction

Erythritol, a four carbon polyol, is a sugar abundant in bovine placental tissues. The ability to catabolyze erythritol preferentially over other sugars by bacteria of the genus *Brucella* has been recognized from long time ago and has been associated to the capability to induce abortions in infected ruminants [1]. Wild type *Brucella* growing in rich medium or in minimal medium supplemented with iron [2] show, when exposed to erythritol, an increased growth after a lag phase. The pathway for erythritol degradation in *Brucella* was elucidated by Sperry and Robertson by using radiolabelled compounds [3] and the genes organized in an operon for erythritol catabolism (*eryABCD*) in *B. abortus* have been identified and characterized in our laboratory [4]. The products of the genes were identified as an erythritol kinase (EryA) [5], two putative dehydrogenases (EryB and EryC) and a repressor (EryD) [4]. *B. abortus* S19 is a spontaneously attenuated strain which has been extensively used for vaccination of cattle against brucellosis during decades, and is still widely used [6]. S19 strain carries a deletion affecting the 3′ end of *eryC* and the 5′ region of *eryD* leading to a fused EryC-D polypeptide [7]. This strain does not induce abortions and it is inhibited by erythritol [8], reinforcing the possible role of erythritol in virulence.

The genome sequence of strain S19 has been determined and compared with two virulent *B. abortus* strains, 2308 and 9–941. Three major differences were described, two of them in the *ery* locus, and a third one in a putative adhesin [9]. The differences found in the *ery* locus were the deletion mentioned above, and a second one affecting a putative sugar transporter identified as an erythritol transporter by homology with *Rhizobium leguminosarum* [10]. Further evidence for the possible involvement of erythritol metabolism in virulence has been suggested by Burkhardt *et al*., who demonstrated that inactivation of the *eryC* gene significantly reduced the intramacrophagic and intramurine fitness of *B. suis* [11]. Moreover, erythritol metabolism is connected in *Brucella* with iron uptake by the production of siderophores 2,3-dihydroxybenzoate (2,3-DHBA) and brucebactin [12],[13]. An *entC* mutant unable to synthesise DHBA was not able to cause abortions in pregnant goats, underscoring the role played by DHBA in the virulence [14]. An *entF* mutant unable to produce brucebactin did not grow in the presence of erythritol under iron limiting conditions suggesting that much more iron is needed for the efficient metabolism of erythritol [2]. On the other hand, vaccine strain S19 does not express the same levels of *virB* genes encoding a type IV secretion system (T4SS) as other *Brucella* strains, including *B. abortus* 2308 [15]. The *virB* operon is essential for virulence and intracellular replication within macrophages and mutants in this system as well as vaccine strain S19 are degraded in phagolysosomes [16].

To provide a better understanding of the role of erythritol metabolism in *Brucella*, global gene expression of virulent strain *B. abortus* 2308 has been evaluated in the presence or absence of erythritol in the growth medium. This has been achieved by a combination of a *Brucella* DNA microarray and RNA-seq. The results of this work show that in the presence of erythritol, *Brucella* experiences profound changes in the metabolism, especially in the central carbon metabolism and amino acid metabolism. As has been recently reviewed by Barbier *et al*. [17], we suggest that the maintenance of a defined energy and metabolic balance is essential for intracellular *Brucella* fitness and henceforth for virulence.

## Materials and Methods

### Bacterial strains, media and chemicals

*B. abortus* 2308, a virulent type strain was grown in *Brucella* broth (BB) or *Brucella* agar (BA) plates (Pronadisa, Spain). *Escherichia coli* strains were grown in LB broth or plates. Unless stated otherwise, chemicals and reagents were obtained from Sigma–Aldrich (St. Louis, MO, USA). Restriction enzymes and DNA modifying enzymes were purchased from Fermentas (USA). Oligonucleotides were synthesized by Sigma–Aldrich.

### Isolation and labelling of mRNA from *Brucella*

*B. abortus* strain 2308 was grown in 10 mL of BB in a 100-mL flask on an orbital shaker (200 rpm) overnight at 37°C. Aliquots of 2 ml of this culture were used to inoculate two new flasks containing 10 ml of fresh BB. Erythritol was added at 1 mg/ml to one of them and both cultures were then allowed to grow during 2.5 h until an OD_600_ = 0.6–0.7 was reached. At this time point no significant difference in growth was observed between both cultures. RNA was purified by using the Qiagen RNeasy Protect bacteria mini kit (Quiagen, USA) with on-column RNase free DNase I digestion for removal of genomic DNA according to the manufacturer's instructions. RNA samples were tested for the lack of *Brucella* genomic DNA contamination by PCR with primers specific for the constitutive IF-1 *Brucella* gene (BAB1_0282 - BMEI1671) [18],[19] after 30 cycles of amplification and checked for the presence of a 220 bp band. DNAse I treatment was repeated until no band was visible by gel electrophoresis, although usually one treatment was sufficient. Samples were assayed for RNA integrity using an Agilent 2100 Bioanalyzer and were quantified on a NanoDrop ND-1000 spectrophotometer (Thermo Scientific, USA). Samples were stored at −80°C until used. About 10 µg of pure RNA were depleted of rRNA with the MICROBExpress Kit (Ambion, USA). *Brucella* mRNA for microarray analysis was amplified by the MessageAmp II-Bacteria kit (Ambion) and an antisense amino-allyl dUTP marked RNA (aRNA) was obtained and labeled with Cy3 fluorescent dye (Amersham Bioscience, USA) following the manufacturer's instructions.

### *Brucella* DNA microarray and Data analysis

A whole-genome DNA microarray based on the *Brucella* ORFeome library (EMBL-EBI ArrayExpress Accession Number E-MEXP-1887) [20] was used for global gene expression analysis as was described in Viadas *et al*. [21],[22]. The microarray experiment design was made according to the MIAME recommendations [23]. Four independent RNA samples for each condition (in the presence or absence of erythritol) representing four biological replicates were used. mRNA obtained from bacteria grown with and without erythritol, and labelled with Cy3, was separately hybridized against the microarray and fluorescence scanned. Fluorescence intensity data were analyzed with BRB array tools [24]. Data were normalized against the median reference array and differential expression was evaluated with the Significance Analysis of Microarrays (SAM) method. Results were also deposited in the EMBL-EBI ArrayExpress with Accession Number E-MTAB-1189.

### RNA-seq and Data analysis

High-throughput RNA-sequencing (RNA-seq) [25] provides an effective way to measure transcriptome data. Preparation of RNA samples for RNA-seq was essentially as described above with several modifications to improve rRNA removal as well as mRNA integrity. Pure RNA (5 µg) was depleted of rRNA with the Ambion MICROBExpress kit. The kit is devised to use 10 µg RNA per reaction, using only half of the kit recommended load capacity we found a significant improvement in rRNA removal. This modification results in a higher percentage of non-rRNA reads in the final result. About 100 ng of this enriched mRNA was used to construct the library for subsequent sequencing.

Libraries were generated using the Illumina mRNA-seq sample preparation kit (Illumina, San Diego, USA) following strictly the manufacturer's instructions. After purification, the mRNA was fragmented into 200–500 nt pieces using divalent cations at 94°C during 5 min. Then, the cleaved RNA fragments were copied to first strand cDNA using SuperScript II reverse transcriptase (Invitrogen, USA) and random hexamer primers. This was followed by second strand cDNA synthesis using DNA polymerase I and RNase H. The quantity and quality of the resulting double-stranded cDNAs was assessed using the Qubit 2.0 fluorometer (Invitrogen).

The cDNA was end repaired by T4 DNA polymerase, Klenow DNA polymerase and T4 polynucleotide kinase. Next, the ends were ligated to the adapters, the ligation reaction was gel purified in 2% agarose and fragments of 250 bp average length were further amplified by PCR using Phusion DNA polymerase (Finnzymes, USA) for 15 cycles to minimize skewing of the library. These cDNA libraries were diluted to 6 pM and hybridized to the flow-cell using a Single-Read Cluster Generation Kit v2 (Illumina). Further amplification of each DNA single molecule was performed by 35 cycles of isothermal amplification in the flow-cell on the Cluster Station. After amplification, one of the strands was removed and the genomic sequencing primer hybridized. The flow-cell was transferred to the Genome Analyzer IIx and sequencing was performed for 36 cycles using SBS Sequencing Kit v3 (Illumina) in the Massive Sequencing Service of the IBBTEC-University of Cantabria.

Raw sequences were aligned to the reference *B. abortus* 2308 genome using the open software package MAQ [26]. In order to count the number of times each base of the reference genome was contained in a single read, we used the Maq pileup utility. From the output of this program we parsed a file containing the complete reads per base of the genome. This file contains a single column of numbers indicating how many times this base was read and a row per base of the genome (3,278,307 rows). Such a file may be used by the visualizing program Artemis [27] as a user plot to indicate graphically the transcription level along the complete genome. We used a perl script to calculate the number of reads crossing each CDS and this number was converted to the standard RPKM (Reads per kilobase of feature and per million mapped reads, rRNA reads excluded) as described [28]. Before comparing between conditions, the two datasets were normalized with respect to the median of the erythritol sample. A feature was considered differentially regulated if displayed at least a 2-fold change in gene expression. The results are deposited in the EMBL-EBI ArrayExpress with Accession Number E-MTAB-1203.

### Quantitative real-time RT-PCR (qRT-PCR)

The data obtained from both the microarray and RNA-seq experiments were further validated by qRT-PCR. cDNA was generated from 2 µg of total RNA using random oligonucleotide hexamers and SuperScript III RT (Invitrogen) according to manufacturer's instructions. About 1 µL of the cDNA template was used in quantitative real-time PCR reactions using Power SYBR® Green PCR Master Mix (Applied Biosystems, USA) and a 7500 Real Time PCR System (Applied Biosystems). Primers were designed using Primer3 (http://primer3.sourceforge.net). To confirm the lack of DNA contamination, reactions without reverse transcriptase were included. Threshold fluorescence was established within the geometric phase of the exponential amplification and the cycle of threshold (Ct) was determined for each reaction. All qRT-PCR reactions were done by triplicate and the constitutively expressed gene IF-1 of *Brucella* was used as an internal control. The 2^−ΔΔCT^ method was used for quantitation [29]. Results were expressed as ΔΔCt = ΔCt _condition 1_−ΔCt _condition 2_; ΔCt _each primer_−ΔCt _IF-1 primers_).

## Results and Discussion

### Microarray gene expression data

**Figure 1:**
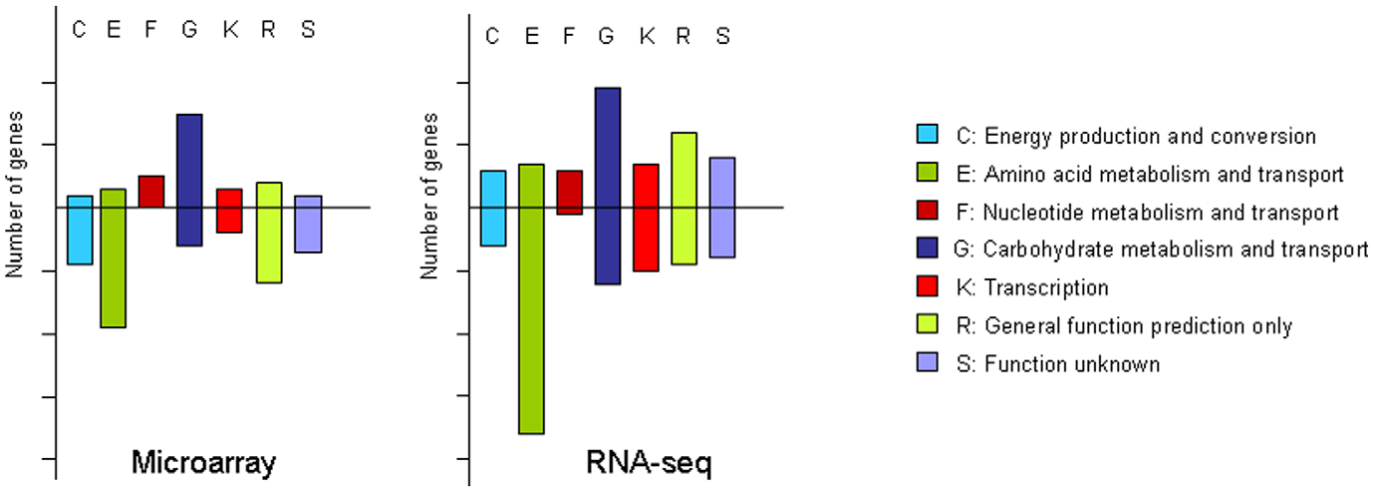
Histogram showing the distribution of regulated genes in COG categories. Bar height is proportional to the number of genes in each category, each vertical mark representing ten genes. Bars above and below the axis represent up- and down-regulated genes, respectively. Categories containing 5 or less regulated genes in each of the experimental approaches have been omitted from the histogram.

To determine the changes in gene expression produced by erythritol in *Brucella*, we used an ORFeome-derived microarray containing most of the CDS's of *Brucella.* This microarray has been recently validated for the study of gene expression in *B. abortus* 2308 [21]. Genes were considered as being differentially expressed when they passed the SAM test with a false discovery rate lower than 0.05. Using this criterion, we found 129 genes whose expression was modified in the presence of erythritol. The products of 117 of these genes, 35 of which were up-regulated and 82 down-regulated, could be associated with a particular Cluster of Orthologous Groups of proteins (http://www.ncbi.nlm.nih.gov/COG/). 55% of these genes were associated with cellular metabolism. The most significant finding was the abundance of genes related with carbohydrate metabolism and transport among the up-regulated genes (15 out of 35) and genes involved in amino acid metabolism and transport among the down-regulated genes (19 out of 82). The complete lists of ORF's with differential expression in the presence of erythritol are shown in Tables S1 and S2, and a summary of up- and down-regulated genes sorted by COG categories is shown in Figure 1.

### Identifying differentially expressed genes by RNA-seq

RNA-seq is an approach that provides tens of millions of short sequence reads from RNA's that can be easily mapped to a reference genome [25]. The *Brucella* transcriptome was determined by RNA-seq using the Illumina GAIIx sequencing platform. We used two cDNA libraries made from enriched mRNA, obtained from *in vitro* cultures grown with and without erythritol. The raw sequence data have been deposited in the Array Express Archive (http://www.ebi.ac.uk/arrayexpress/) database and the metrics of the reads are summarized in Table 1. We used MAQ [26] to align the reads (35 nt long) with the *B. abortus* 2308 reference genome (PRJNA16203) allowing two mismatches. Performance of the sequencing run was better in the sample obtained with erythritol resulting in a higher number and quality of reads. mRNA enrichment was partial, as only about 35% of the aligned reads corresponded to mRNA's, but we still obtained 4 and 3 million of 35 nt long non rRNA reads from the BB with erythritol and the BB samples, respectively. While the ORFeome microarrays provide only data for the included ORF's, RNA-seq gives a continuous value for transcription along the complete genome. This signal can be integrated for any genome feature defined by two coordinates. The transcription level was computed for all CDS's annotated in the *B. abortus* 2308 genome. We observed 262 genes with their expression modified more than twofold in the presence of erythritol, 86 of them had not an assigned COG category, and encoded products smaller than 100 amino acids. Among the 176 genes with an assigned COG, 83 were up- and 93 down-regulated. Genes involved in metabolism were again the most represented, particularly those involved in carbohydrate metabolism and transport among the up-regulated (19 out of 83) and the implicated in amino acid metabolism and transport among the down-regulated (36 out of 93). (Figure 1 and Tables S1 and S2). The number of regulated genes identified was lower in the array experiment than in the RNA-seq. This was due to the more stringent conditions used to detect differentially expressed genes with the microarrays. We found a strong coincidence in the set of up-regulated genes with 26 coincident genes out of the 35 identified in the microarray experiment. The coincidence was lower in the set of down-regulated genes. It also should be noted that we never obtained contradictory results for any gene in the two approaches used.

**Table 1:** Summary of the RNA-sequencing data.

	BB+erythritol	BB
Total reads	16,064,214	13,835,287
Aligned reads (maq)	13,073,703 (81.4%)	8,762,781 (63.3%)
rRNA	9,024,569	5,731,599
Unique	4,049,134	3,031,182

In order to obtain an insight on the biological meaning behind the lists of erythritol regulated genes we have analyzed their functional capabilities according the annotations available in the COG database and the KEGG Orthology system (http://www.genome.jp/kegg/ko.html). The KEGG pathway maps (http://www.genome.jp/kegg/pathway.html) have also been used to ascribe the participation of identified genes in the pathways present in *B. abortus* 2308. This analysis has selected a group of genes whose changes in expression level were easily integrated in a biological response to erythritol that is detailed in the next sections. These selected genes are shown in Table 2.

**Table 2:** *B. abortus* genes regulated by erythritol selected by their biological significance.

ORF	Array^a^	RNAseq^b^	Annotation	COGs^c^
BAB2_0364	2.51	2.78	Fructose-1,6-bisphosphatase	COG0158G
BAB2_0365	2.72	3.56	Fructose-1,6-bisphosphate aldolase	COG0191G
BAB2_0366	5.35	9.23	Ribose-5-phosphate isomerase B	COG0698G
BAB2_0367	2.64	7.75	Triose phosphate isomerase	COG0149G
BAB2_0368	2.24	5.74	DeoR family regulatory protein	COG1349GK
BAB2_0369	2.78	2.09	Erythritol repressor, EryD	COG2390GK
BAB2_0370	2.38	3.29	Erythrulose 4-phosphate dehydrogenase, EryC	-------------G
BAB2_0371	2.05	3.08	Erythritol 4-phosphate dehydrogenase, EryB	COG0578G
BAB2_0372	2.58	3.85	Erythritol kinase, EryA	COG1070G
BAB2_0375	2.78	2.23	Phosphoribulokinase ABC transporter ATPase	COG1129G
BAB2_0376	1.96	2.38	Inner-membrane translocator	COG1172G
BAB2_0377	2.16	2.05	Periplasmic protein/LacI transcrip. regulator	COG1879G
BAB2_0378	-	2.30	DeoR family regulatory protein	COG1349GK
BAB1_1741	1.59	-	Glyceraldehyde 3-phosphate dehydrogenase	COG0057G
BAB1_1742	1.75	2.48	Phosphoglycerate kinase	COG0126G
BAB1_0448	-	2.20	Phosphoglycerate/bisphosphoglycerate mutase	COG0406G
BAB1_1813	2.08	2.00	Putative translaldolase	COG0176G
BAB2_0491	0.55	0.47	Extracellular solute-binding protein	COG1653G
BAB2_0547	0.61	0.48	Solute-binding family 1 protein	COG1653G
BAB2_0548	0.67	0.34	Vacuolar H+-transporting ATPase	COG1175G
BAB2_0712		0.40	Branched chain ketoacid dehydrogenase	COG1249C
BAB2_0713	0.57		Branched chain ketoacid dehydrogenase	COG0508C
BAB2_0714	0.53	0.44	Branched chain ketoacid dehydrogenase	COG0022C
BAB2_0715		0.46	Branched chain ketoacid dehydrogenase	COG1071C
BAB1_0977	-	2.29	Fumarate lyase	COG1951C
BAB1_1927	1.81	-	Malate dehydrogenase	COG0039C
BAB2_0863	-	0.22	Glutaminase	COG2066E
BAB2_0865	0.63	0.34	Pyridoxal-dependent decarboxylase	COG0076E
BAB2_0866	0.46	0.39	Glutamate decarboxylase alpha	COG0076E
BAB1_1792	0.59	0.23	Leu/Ile/Val-binding family protein	COG0683E
BAB1_1794	0.56	0.38	Leu/Ile/Val-binding family protein	COG0683E
BAB2_0282	0.55	0.33	Leu/Ile/Val-binding family protein	COG0683E
BAB1_1697	2.67	7.98	D-3-phosphoglycerate dehydrogenase	COG0111EH
BAB1_1699	-	5.80	Phosphoserine aminotransferase	COG1932EH
BAB1_1502	2.15	2.57	Carbamoyl phosphate synthase small subunit	COG0505EF
BAB1_1508	2.11	2.54	Carbamoyl phosphate synthase large subunit	COG0458EF
BAB2_0640	1.76	2.30	Dihydroorotase	COG0044F
BAB2_0641	1.71	2.94	Aspartate carbamoyltransferase catalytic sub.	COG0540F
BAB1_1824	1.56	2.61	PurH	COG0044F
BAB1_0355	0.35	0.32	Murein hydrolase exporter	COG1380R
BAB1_0356	0.34	-	Murein hydrolase export regulator	COG1346RM
BAB1_1219	13.45	20.62	Hypothetical protein (Protease I)	COG0693R
BAB1_1220	4.86	4.66	Hypothetical protein (intrac. protease)	COG4321R
BAB1_1696	1.53	2.56	GCN5-related N-acetyltransferase	COG0456R

a Fold-change microarray;

b Fold-change RNA-seq;

c The COG number and functional categories: C: Energy production and conversion; E: Amino acid metabolism and transport; F: Nucleotide metabolism and transport; G: Carbohydrate metabolism and transport; H: Coenzyme metabolism; I: Lipid metabolism; J: Translation; K: Transcription; M: Cell wall/membrane/envelope biogenesis; R: General function prediction only, S: Function unknown.

### Validation by PCR

A set of genes associated and not associated with metabolism were selected for further validation by qRT-PCR as described in Materials and Methods using the primers indicated in Table S3. The genes were chosen according to the different expression levels under erythritol treatment. We selected some representatives among the up- and down-regulated genes, and also genes expected to form part of a regulated operon. In all the cases the over-expression or repression was confirmed (Table 3).

**Table 3:** Real-time PCR confirmation of selected ORFs with significant changes in mRNA expression under erythritol treatment.

ORF	Annotation	2^−ΔΔCT^
BAB1_1129	Histone- like DNA- binding protein	4.20
BAB1_1219	Hypothetical protein (Protease I)	12.20
BAB1_1473	Trans-aconitate 2-methyltransferase	7.95
BAB1_1502	Carbamoyl phosphate synthase small subunit	3.65
BAB1_1508	Carbamoyl phosphate synthase large subunit	2.20
BAB1_1697	D-3-phosphoglycerate dehydrogenase	3.30
BAB1_1699	Phosphoserin aminotransferase	4.65
BAB1_1813	Putative transaldolase	4.40
BAB2_0364	Fructose-1,6-bisphosphatase	4.15
BAB2_0367	Triosephosphate isomerase 2 (TIM 2)	8.05
BAB2_0371	Erythritol-4-phosphate-dehydrogenase	9.25
BAB2_0375	Phosphoribulokinase ABC transporter ATPase	3.60
BAB1_0355	LrgA family protein (murein hydrolase exporter)	0.20
BAB1_0356	LrgB-like protein (murein hydrolase exporter regulator)	0.50
BAB1_1792	Leu/Ile/Val-binding family protein	0.35
BAB1_1855	GCN5-related N-acetyltransferase	0.19
BAB1_1856	GCN5-related N-acetyltransferase	0.23
BAB2_0282	Leu/Ile/Val-binding family protein	0.32
BAB2_0518	Proline dehydrogenase	0.70
BAB2_0873	Hypothetical protein	0.75

### Genes related with carbohydrate metabolism

**Figure 2:**
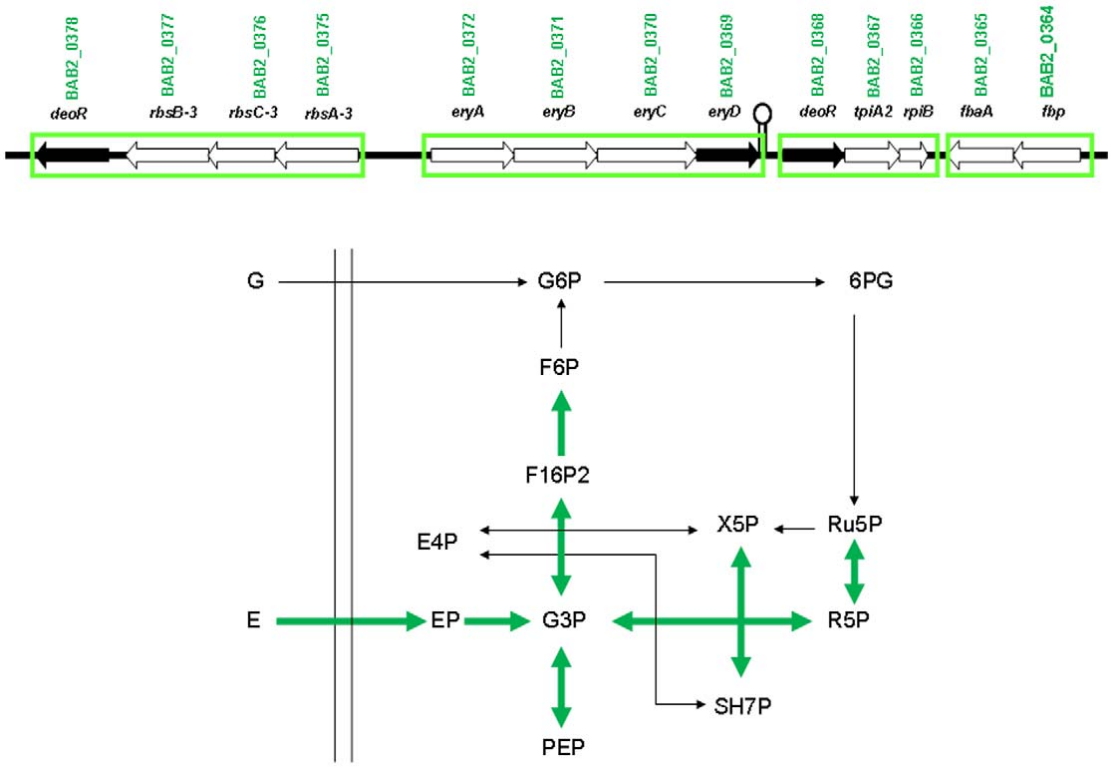
Effect of erythritol on carbohydrate metabolism. Genetic organization of the cluster of genes surrounding the *ery* operon involved in catabolism of erythritol, represented at the top of the Figure. Below, the diagram shows the most significant steps regulated by erythritol in the carbohydrate metabolism pathways. Thick arrows indicate those reactions catalyzed by up-regulated enzymes.

Among 34 differentially regulated genes in the carbohydrate metabolism category, 19 were up-regulated. Most of them are found at the *ery* locus, where they defined a cluster of 13 contiguous genes (BAB2_0364-BAB2_0378), organized in at least three transcriptional units that showed coordinate expression mediated by erythritol (Figure 2). In the center of this cluster, we find the *ery* operon, *eryABCD* (BAB2_0372-BAB2_0369), involved in catabolism of erythritol. Upstream *eryABCD*, and transcribed from the opposite strand, there is a cluster of 4 genes (*rbsACB deoR*, BAB2_0375-BAB2_0378) annotated in *Brucella* as a possible ribose transporter, and including a DeoR-family regulator. Three more genes (*deoR tpiA2 rpiB*, BAB2_0368-BAB2_0366) immediately downstream *eryABCD* encode another DeoR-family regulator and two sugar phosphate isomerases. Finally, two genes (*fbp fbaA*, BAB2_0364-BAB2_0365), transcribed from the opposite strand, encode two enzymes involved in glycolysis/gluconeogenesis: fructose-1,6-bisphosphatase and fructose-1,6-bisphosphate aldolase. The erythritol catabolic pathway was elucidated by Sperry and Robertson [3], and converts erythritol into dihydroxy acetone phosphate (DHAP) through an initial phosphorylation step followed by three dehydrogenation steps and a final decarboxylation. EryABCD constitute the erythritol catabolic pathway, where EryA is the erythritol kinase, EryB is the erythritol phosphate dehydrogenase, and EryD is a regulator that induces the operon in the presence of erythritol. EryC has been hypothesized to be the D-erythrulose-1-phosphate dehydrogenase [4]. The very consistent up-regulation mediated by erythritol, the physical proximity between the genes, and previous results reported by Yost *et al.* [10] in the closely related alphaproteobacteria *Sinorhizobium meliloti* suggest that the putative ribose transporter found upstream *eryABCD* would actually be an erythritol transporter, as pointed out in Crasta *et al*. [9]. The fact that in *B. abortus* strain S19 the BAB2_0376 ortholog (BAbS19_II03540) is affected by a 68 bp deletion and probably inactivated suggests the existence of alternative erythritol uptake systems in *Brucella*. The end product of erythritol catabolism would be DHAP, which is converted into glyceraldehyde-3P (GA3P) by the enzyme triose phosphate isomerase (TPI). *Brucella* genome contains two *tpi* genes, BAB1_1161 and BAB2_0367 that is regulated by erythritol. *S. meliloti* also contains two genes encoding functional triose-phosphate isomerases with distinct biochemical roles in the organism. TpiA is necessary for glycerol catabolism, whereas TpiB is necessary for the catabolism of erythritol [30]. BAB2_0367, by synteny with other alphaproteobacteria and mode of regulation, would probably be the *Brucella*, ortholog of *S. meliloti* TpiB, the erythritol pathway dedicated enzyme. BAB2_0367 (*tpi*) together with BAB2_0366 (*rpi*) and BAB2_0368 (*deoR*) may either form a transcriptional unit by themselves or be part of the *eryABCD* operon. The distance between *eryD* and *deoR* is only 88 bp and while the observed co-regulation with *eryABCD* argues in favor of the integration in the *ery* operon, the presence of an independent regulator, as well as the presence of a putative transcriptional terminator in the intergenic region, argue in favor of the existence of an independent transcriptional unit. The last two genes in this cluster, BAB2_0364 and BAB2_0365, which would also form an independent transcriptional unit, encode two enzymes involved in glycolysis and gluconeogenesis: fructose-1,6-bisphosphatase and fructose-1,6-bisphosphate aldolase. The association of the genes for this two enzymes is very interesting since *Brucella* is known to lack the enzyme phosphofructokinase, and the only way to produce fructose-1,6-bisphosphate (F1,6BP) is the reaction catalyzed by fructose-1,6-bisphosphate aldolase (BAB2_0365). The co-expression of fructose-1,6-bisphosphatase can produce the rapid conversion of F1,6BP into fructose-6P (F6P) pulling the equilibrium of these two reactions in the gluconeogenic direction. As mentioned with TPIs, there are also two different fructose 1,6- bisphosphatases in *Brucella* (BAB1_1292 and BAB2_0364) and only BAB2_0364 is induced by erythritol. This suggest again that this fructose-1,6-bisphosphatase plays an specific role in erythritol metabolism.

Other up-regulated genes related to carbohydrate metabolism, but mapping outside the *ery* cluster were *gap* and *pgk* (BAB1_1741-BAB1_1742), which may form an operon and BAB1_0448. The products of these genes, namely glyceraldehyde 3-phosphate dehydrogenase (Gap), phosphoglycerate kinase (Pgk) and phosphoglycerate mutase (BAB1_0448), catalyze three consecutive reactions in the transformation from GA3P to pyruvate in the final steps of glycolysis. Finally BAB1_1813 encodes the transaldolase that operates in the nonoxidative phase of the pentoses phosphate shunt (Figure 2).

In addition to the genes participating in sugar metabolism we found two up-regulated genes encoding the enzymes of the tricarboxylic acid cycle (TCA) fumarate lyase (BAB1_0977) and malate dehydrogenase (BAB1_1927). These two enzymes catalyze consecutive steps of the TCA cycle that interconvert fumarate and oxaloacetate. Overexpression of these genes by erythritol suggest an increased level of oxaloacetate that together with the elevated flow in lower glycolysis may produce an increment of the amount of Acetil CoA that enters TCA, thus providing the necessary energy boost needed for the subsequent increased growth.

On the other hand, we found 3 down-regulated genes, BAB2_0491, BAB2_0547 and BAB2_0548, (Table S2), that are putatively involved in sugar transport systems. This could indicate that erythritol up-regulates its own transport while down-regulates the transport of other sugars. When *Brucella* grows in the presence of both erythritol and glucose, it has been observed that the uptake of glucose is not affected, and the incorporation of glucose into metabolic carbon flux is reduced by 30% [31]. This is in agreement with the observation that BAB2_0184, the Glucose/Galactose transporter found in *Brucella* [32] does not appear to be regulated by erythritol in our experiments. The observed reduction of 30% in the incorporation of glucose into metabolic carbon would be explained by the additional supply of F6P through the activity of the erythritol induced genes. It is also possible that the incorporation of other sugars is reduced by the down regulation of their transporters.

Glucose metabolism in *Brucella* is mainly carried out through the pentose phosphate pathway since glycolysis is blocked by the lack of phosphofructokinase. The nonoxidative phase of the route allows interconversion between sugars of 3 to 7 carbon atoms as shown in Figure 2. The results presented here show that erythritol induced genes involved in his own transport and degradation to GA3P. This effect is complemented by induction of genes not directly related to erythritol transport or degradation. Increased expression of the genes for glycolytic and gluconeogenic enzymes, for enzymes of the nonoxidative phase of the pentose phosphate pathway and for some TCA enzymes may result in an efficient cycle that converts in each round a molecule of F6P in ribulose-5P (Ru5P) with liberation of CO_2_ and the recovery of energy in the form of reducing power. Glucose uptake, in agreement with previously described results, would not be affected, and the transport of other sugars could be reduced also, explaining the well known preferential use of erythritol over other substrates.

### Genes related with amino acid and nucleotide metabolism

**Figure 3:**
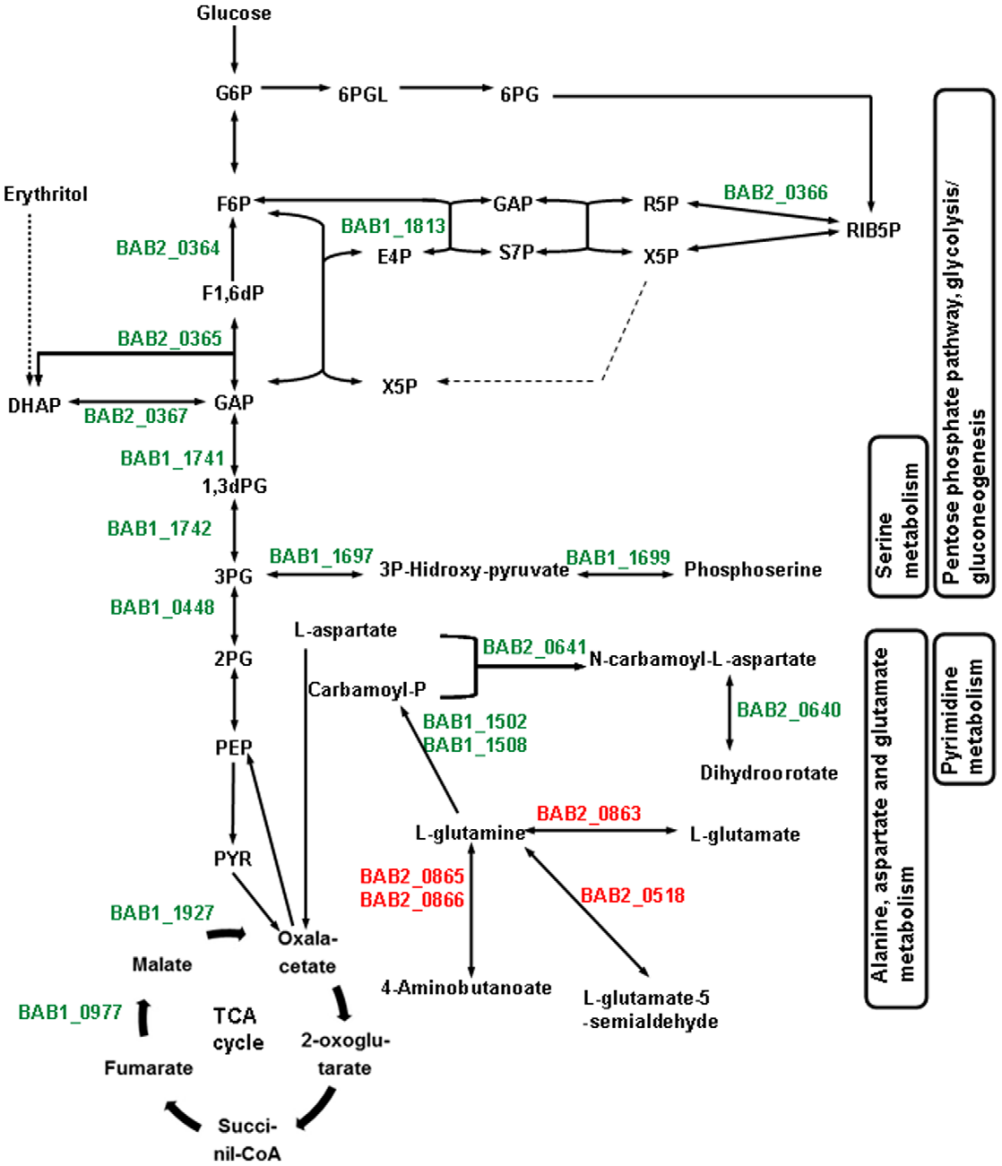
Map of the main carbohydrate and amino acid metabolic pathways regulated by erythritol in *B. abortus* 2308. Genes up-regulated are shown in green and genes down-regulated are shown in red. Adapted with permission from Barbier *et al* [17].

Only 6 out of 41 genes involved in aminoacid metabolism and transport were consistently up-regulated. BAB1_1697 (*serA*, D-3-phosphoglycerate dehydrogenase) and BAB1_1699 (*serC*, phosphoserine aminotransferase), together with BAB1_1696 (N-acetyltransferase-GCN5) which does not belong to this functional category, form an up-regulated operon involved in the transformation of 3-phosphoglycerate into 3-phosphoserine during serine biosynthesis. The N-acetyltransferase-GCN5 is a putative lysine acetylase that could regulate metabolic enzyme activity. This cluster could be diverting part of the glycolytic flux to phosphoserine and then to serine and other amino acids. BAB1_1502 and BAB1_1508, which encode for both the small and large carbamoyl phosphate synthase subunits, are also up-regulated. This enzyme produces carbamoyl phosphate, a key metabolite for the biosynthesis of amino acids and pyrimidines. Two amino acid families, alanine/aspartate/glutamate and arginine/proline have carbamoyl phosphate in the start of their biosynthetic pathways. On the other hand, aromatic amino acids and catecholic siderophores may be synthesized from the pentose pathway intermediate erythrose-4P. In summary, most of the amino acids may have their biosynthesis up-regulated by erythritol (Figure 3).

35 out of 41 of the erythritol-regulated genes involved in aminoacid metabolism and transport were down regulated. Among them, there are at least 15 genes involved in transport systems for different aminoacids, specially ABC transporters for branched-chain amino acids. Most of the remaining genes are associated with amino acid catabolism (Figure 1, Table S2). Among these, the cluster BAB2_0712-0715, encode a branched-chain alpha-keto acid dehydrogenase complex involved in the degradation of this group of aminoacids. According to this, a second deep metabolic effect of erythritol may be to turn up the biosynthesis and turn down the uptake and degradation of amino acids.

Furthermore, BAB2_0640 and BAB2_0641, encoding the two first enzymes in the biosynthesis of pyrimidines: dihydroorotase and aspartate carbamoyl transferase, respectively, are also up-regulated. Since the production of carbamoyl phosphate may be also increased by up-regulation of carbamoyl phosphate synthase, biosynthesis of pyrimidines is probably stimulated by erythritol. Up regulation of BAB1_1824, encoding a bifunctional phosphoribosylaminoimidazolecarboxamide formyltransferase/IMP cyclohydrolase responsible of the biosynthesis of IMP, also indicates an stimulation of the biosynthesis of purines and that would be in accordance with the reported stimulation of growth induced by erythritol [31].

### Virulence and the adaptation to intracellular growth

Erythritol has been linked to virulence in *Brucella* since it was described to be the cause of localization of *B. abortus* in the placenta of pregnant cows [1]. The attenuated vaccine strain S19 carries a deletion in the *ery* operon that has been associated with its attenuation in pregnant cattle [3] [33] but this deletion is not the cause of its attenuation in the mouse model of infection [34]. On the other hand, mutants in *eryB* and *eryC* have shown an attenuated phenotype in cultured macrophages and mice [35].

When comparing our results with those analyzing the intracellular response of *Brucella*, it is noteworthy that the different datasets do not overlap significantly. As an example, only 39 out of the 273 *B. melitensis* genes whose expression changed intracellularly reported by Eskra *et al.* [36], are regulated by erythritol, some showing even different patterns: while 12 genes show the same response in both experiments, 27 show the opposite behaviour. Moreover, functional analysis of the products of those genes does not reveal any obvious biological meaning. Comparison of the erythritol dataset with the intracellular proteome shows a similar result. The most remarkable coincidence was found with the *B. suis* genes up-regulated after 24 hours on intramacrophagic growth identified by a proteomic analysis [37]. We identified coincidences in the up regulation of glycolytic (BAB1_1741 and BAB1_1742), TCA (BAB1_0977 and BAB1_1927) and gluconeogenic genes, as well as aminoacid (BAB1_1697), purines (BAB1_1824) and pyrimidine (BAB1_1502) biosynthetic genes. This coincidence may reflect the similarity in the mechanisms underlying both, the growth stimulation induced by erythritol and the active growth that *Brucella* experiments in macrophages after the initial death phase. A large set of genes related to the translation apparatus induced in *Brucella* growing actively in macrophages and the virulence associated *virB* genes do not appear induced after a 2.5 h exposure to erythritol. This difference may be explained because our reference control without erythritol was already growing actively in rich medium and the macromolecular biosynthesis apparatus was fully expressed. Virulence genes like *virB* need additional signals for induction and their expression was not induced in our experimental condition.

Mutations in several of the genes regulated by erythritol have been shown to produce attenuation to *Brucella* in different virulence models. Some of them are related with carbohydrate and aminoacid metabolism and transport. Thus, mutations in two genes of the erythritol degradative operon, *eryB* and *eryC* (BAB2_0371 and BAB2_370) have been described to produce attenuation. Furthermore, mutants in the phosphoglycerate kinase gene (BAB1_1742) are also attenuated and produced in mice a protection superior to S19 vaccine [38]. Other coincident genes are related to aminoacid metabolism and are closely associated with the transformations affecting glutamine and henceforth to the connexion between the metabolism of carbon and nitrogenated compounds. This connection may also extend to genes involved in cell-wall turnover regulation. Genes BAB1_0355 and BAB1_0356 which are down regulated by erythritol, encode an operon homologous to *Staphylococcus aureus lrgAB*. These genes are involved in regulation of murein hydrolase activity and consequently in cell lysis and antibiotic tolerance. An *S. aureus lrgAB* mutant, as well as some mutants with reduced *lrgAB* expression, exhibited increased lysis and decreased antibiotic tolerance [39]. The function of these two genes in *Brucella* is not known but it has been reported that a mutant with a deleted BAB1_0355 gene was overgrown 100 times by the wild type in the spleen of infected mice [40].

From these comparisons we can conclude that some similarities exist between the metabolic status induced by erythritol and the situation needed for *Brucella* intracellular survival, and that this metabolic adaptation is also crucial to virulence.

The preceding discussion included barely one fourth of the 250 genes differentially regulated by erythritol. Many of unmentioned genes either do not have a function assigned or their contribution to erythritol regulation could not be easily interpreted. As an example, BAB1_1219 and BAB1_1220 were the genes more induced in both the RNAseq and the array experiments. They are annotated as hypothetical proteins and some hints signal that they may be intracellular proteases. However, we cannot provide an explanation on the meaning of their induction.

### How are these gene sets coordinately regulated by erythritol?

There are around 250 genes (with an assigned COG category) reported in this study that see their expression affected in one way or another as a result of the presence of erythritol, representing approximately 100 transcriptional units. Since these changes occurred within 2.5 hours, they represent the early response of *B. abortus* to erythritol. The only regulator currently described to respond to erythritol is EryD. If EryD is regulating directly all the transcriptional units that we have observed in this work, we should be able to align their promoter regions, and identify an “EryD-box”. We have extracted sequences 500 bp upstream from most of these transcriptional units and tried to identify in them a putative EryD operator without a satisfactory result (data not shown). This could indicate that only a fraction of these genes are regulated directly by EryD, or that the putative EryD box does not show high sequence conservation. There are also several regulatory proteins among the regulated genes, that could be responsible of the secondary or indirect response to erythritol.

Since most of the changes reported here involve metabolism we can imagine that global metabolic control in *Brucella* should also be implicated in the control of erythritol induced changes. The best characterized metabolic control system in *Brucella* is the PTS system, consisting of a phosphorelay regulated by the levels of F1,6BP and phosphoenolpyruvate [41]. Since these two metabolites may be increased by erythritol, the *Brucella* PTS could also be involved in the regulation of the erythritol response.

In a recent review, Barbier *et al.* summarize the current evidence linking metabolism and virulence [17]. They suggest that the fine-tuning of *Brucella* metabolism seems to be crucial during host infection, and a key player linking both metabolism and virulence would be the PTS system, by sensing the metabolic state of the cell and probably through the cross-talk with the two-component system BvrS/R. This would in turn regulate the expression of the quorum sensing regulator VjbR, that would be responsible for activation of determinants of virulence like VirB or flagella [17]. This could explain why an *eryC* mutant is avirulent, or why *B. abortus* S19 does not express *virB* the same way that *B. abortus* 2308 does. A time course study of the transcriptional changes induced by erythritol should provide answers to some of the still open questions in the role of erythritol in the virulence of *Brucella*.

## Supporting Information

Table S1 **ORFs of** ***B. abortus*** **with increased levels in mRNA expression in the presence of erythritol.** ORFs with an assigned COG category obtained from microarray and/or RNA-seq experiments indicating increased levels of mRNA in the presence of erythritol, are showed. Fc-array indicates the fold-change of the microarray data. Fc-RNA-seq indicates the fold-change measured by RNAseq. COG number and functional categories are as follows: C: Energy production and conversion; E: Amino acid metabolism and transport; F: Nucleotide metabolism and transport; G: Carbohydrate metabolism and transport; H: Coenzyme metabolism; I: Lipid metabolism; J: Translation; K: Transcription; M: Cell wall/membrane/envelope biogenesis; R: General function prediction only, S: Function unknown.
(XLS)

Table S2 **ORFs of** ***B. abortus*** **with decreased levels in mRNA expression in the presence of erythritol.** ORFs with an assigned COG category obtained from microarray and/or RNA-seq experiments indicating increased levels of mRNA in the presence of erythritol, are showed. Fc-array indicates the fold-change of the microarray data. Fc-RNA-seq indicates the fold-change measured by RNAseq. COG number and functional categories are as follows: C: Energy production and conversion; E: Amino acid metabolism and transport; F: Nucleotide metabolism and transport; G: Carbohydrate metabolism and transport; H: Coenzyme metabolism; I: Lipid metabolism; J: Translation; K: Transcription; M: Cell wall/membrane/envelope biogenesis; R: General function prediction only, S: Function unknown.
(XLS)

Table S3 **Sequence of primers for selected genes used in real-time PCR.** Sequence of the different oligonucleotides used in real-time PCR are indicated.
(XLSX)
